# Effects of orthoptic therapy in children with intermittent exotropia after surgery: study protocol for a randomized controlled trial

**DOI:** 10.1186/s13063-022-06246-4

**Published:** 2022-04-11

**Authors:** Meiping Xu, Fuhao Zheng, Yiyi Peng, Chunxiao Wang, Jiangtao Lou, Huanyun Yu, Yuwen Wang, Xinping Yu

**Affiliations:** 1 Eye Hospital and School of Ophthalmology and Optometry, Wenzhou Medical University, Wenzhou, Zhejiang China; 2 National Clinical Research Center for Ocular Diseases, Wenzhou, Zhejiang China; 3 State Key Laboratory of Ophthalmology, Zhongshan Ophthalmic Center, Sun Yat-sen University, Guangzhou, China

**Keywords:** Intermittent exotropia, Orthoptic therapy, Suboptimal surgical outcome, Study protocol, Randomized controlled trial

## Abstract

**Background:**

Intermittent exotropia (IXT) is the most common type of exotropia in China. Surgery is usually required to align the eye deviation to maintain or obtain better binocular visual function. However, there is a high rate of exodrift or recurrence in surgically treated patients. Orthoptic therapy is sometimes recommended for IXT patients after surgery. However, there is a lack of high-quality randomized controlled trials to prove that orthoptic therapy could be an effective supplement to surgical treatment for IXT patients. The main purpose of this study is to test the clinical effectiveness of orthoptic therapy in long-term stabilization of postoperative IXT patient. This report describes the design and methodology of the Intermittent Exotropia Postoperative Treatment Clinical Trial, which is the first large-sample, blank-controlled, randomized clinical trial.

**Methods:**

A total of 136 IXT patients (aged 7 to 17 years) will be enrolled and assigned to the orthoptic therapy group or blank control group according to a simple randomization scheme. Patients in the orthoptic therapy group will receive at least 2 months of orthoptic therapy, such as anti-suppression, vergence, and accommodation training. Patients in the blank control group will receive only refractive correction. All enrolled patients will need regular follow-up observation until 24 months after surgery. The primary outcome will be the proportion of participants meeting suboptimal surgical outcomes in this 24-month follow-up, which is defined as (1) exodeviation of 10 prism diopters (PD) at distance or near using the simultaneous prism and cover test (SPCT) or (2) loss of 2 or more octaves of stereoacuity from baseline, at any masked follow-up visit examination. The secondary outcomes will be the exodeviation at distance and near using the simultaneous prism and alternate cover test (PACT), magnitude of fusional convergence, stereoacuity, and accommodation. Measurements will be taken at baseline and at the 6-, 12-, 18-, and 24-month follow-ups.

**Discussion:**

To the best of our knowledge, this will be the first prospective, randomized controlled study of orthoptic training in IXT patients after surgery. The aim of this work is to confirm the efficacy of orthoptic therapy in reducing the proportion of recurrence among IXT patients after surgery and improving binocular vision function.

**Trial registration:**

Chinese Clinical Trial Registry ChiCTR1900026891. Registered on 25 October 2019.

## Background

Intermittent exotropia (IXT) is the most common type of childhood-onset exotropia [1, 2]. At the same time, IXT is also the most common type of strabismus in China, and its incidence is approximately 3.26% [3]. IXT patients usually present exodeviation when they are fatigued, distracted, or looking at distance, and the alignment of the eye position is controlled when they are nervous or focused. With the progression of disease, there is a worse control of exodeviation, which leads to deterioration in binocular vision and negative psychosocial consequences [4, 5]. In such cases, surgery is often the treatment of choice. A recent study also shows that IXT is the main contributor to increase in strabismus surgery in China [6]. However, whether surgery benefits long-term ocular alignment in IXTs is unclear, as a high recurrence rate has been recently reported [7–10]. The most important factor determining surgical outcomes is the duration of follow-up. With extensions in follow-up time, the success rate of surgery shows a decreasing trend [11]. Kim et al. revealed that more than 50% of the total amount of exodrift occurred within the first post-operation year [12], and several other studies have reported that the main recurrence of exodeviation occurred within 2 years after surgery [13–15].

The main objective of orthoptic therapy for IXTs is to remove suppression, correct abnormal retinal correspondence, improve sensory fusion and fusional reserve, and obtain better binocular vision. Previous studies have mainly focused on patients with small deviation IXT to obtain better fusional control [16, 17]. The usage of surgery coupled with pre- or postsurgical, or both, nonsurgical treatments, including orthoptic training and occlusion therapy, has been advocated by different ophthalmologists around the world [18–21], and such assessments have been based on clinical impressions rather than solid evidence. We have conducted a survey about the clinical options on non-surgical management of IXTs in China. In total, 56% (271/488) participants are considered orthoptic treatment as the most effective non-surgical intervention [22]. So, there is an urgent need for randomized clinical trials to validate the effectiveness of orthoptic exercises and further to establish treatment guidelines accordingly. The primary objective of this study was to confirm whether orthoptic therapy could improve the long-term stability of eye position in IXT patients after surgery. Secondly, we wanted to find to what extent could the binocular function be improved by orthoptic training, since our previous study shows that IXTs had subnormal binocular function (e.g., stereopsis, fusional convergence reserve) after surgery [23], and the key indexes of binocular function could be related to the long-term stability. Therefore, we have designed a randomized, controlled trial including two groups: an orthoptic therapy group and a control group to explore the clinical efficacy of orthoptic training in IXTs after surgery over a 2-year period.

## Methods

### Study design

**Fig. 1:**
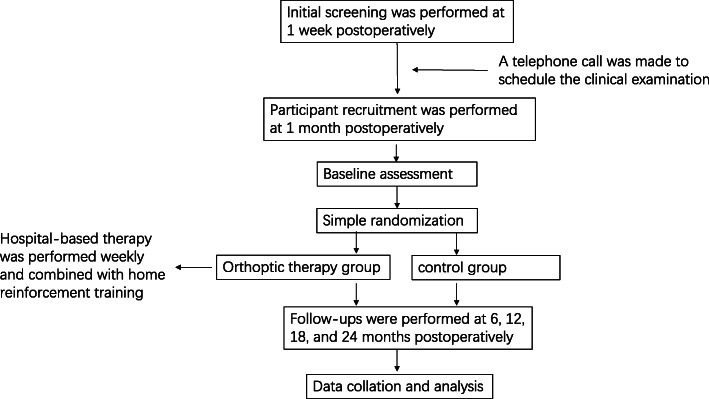
Flowchart of our current study

This will be a prospective, parallel, simple randomized controlled trial. All procedures meet the tenets of the Declaration of Helsinki and were approved by the medical ethics committee of the Affiliated Eye Hospital of Wenzhou Medical University (2019-108-K-101). Written consent and verbal assent will be obtained from parents (or guardians) and participants, respectively. The study protocol (V2/2019.08.10) was registered in Chinese Clinical Trial Registry (ChiCTR1900026891), which is granted for public access and is in accordance with the Standard Protocol Items: Recommendations for Clinical Interventional Trials (SPIRIT) guidelines [24]. The study will be performed in the Affiliated Eye Hospital of Wenzhou Medical University. The research flowchart is shown in Fig. 1. In the case of any research protocol changes during the trial, the project coordinator will submit the appropriate documentation to the ethics board (the medical ethics committee of the Affiliated Eye Hospital of Wenzhou Medical University) and request for modifications. Upon approval, any changes will be notified to relevant investigators, and the trial registry will be updated accordingly.

### Recruitment

All participants will be recruited from an outpatient clinic. We asked surgeons to introduce the trial to prospective patients at the follow-up time of 1-week after surgery. The patients will be informed about the objectives, approaches, advantages, possible adverse effects, and management of this trial. For patients who are willing to participate in this project, the investigators will schedule an appointment for a 1-month postoperative review. To ensure adequate participant enrolment, we will also post the recruitment information in hospital areas (e.g., outpatient hall, inpatient ward), and on WeChat, a social media platform.

### Participant screening

Two well-trained investigators in the amblyopia and strabismus department will be responsible for the recruitment and management of participants. Those who are willing to participate and meet the eligibility criteria will be informed of the basic information related to and the requirements of the project. The inclusion and exclusion criteria are listed in Table 1.

**Table 1:** Inclusion and exclusion criteria

Inclusion criteria	
1.	Patient is 7–17 years old.
2.	Amount of exodeviation measured using PACT at 1 month after IXT surgery meets any of the following:
	2.1 Exodeviation < 10 PD at near and distance.
	2.2 Ortho at near, and exodeviation < 10 PD at distance.
	2.3 Ortho at distance, and exodeviation < 10 PD at near.
3.	Best corrected visual acuity (BCVA) in the worse eye is 0.1 logMAR or better and there has been no previous diagnosis or treatment for amblyopia.
4.	Participant wore spectacles for at least 1 week if refractive error meets any of the following:
	4.1 Myopia< − 0.50 D and > − 6.00 D SE in either eye.
	4.2 Anisometropia < 1.50 D SER.
	4.3 No hyperopia of more than + 3.50 D SER in either eye.
5.	Refractive correction for participants meeting the above refractive error criteria must meet the following guidelines:
	5.1 For myopia, the intent is to fully correct.
	5.2 For hyperopia, the spherical component can be reduced at investigator discretion as the principal of maximum plus to maximum visual acuity.
	5.3 For astigmatism, cylinder must be within 0.25 D of full correction and axis must be within 5° of full correction.
	5.4 Myopic participants should not take any intervention for myopia control, such as orthokeratology, peripheral defocus lenses, and low-concentrate atropine.
6.	No atropine was used within the previous month.
7.	Patient had gestational age > 34 weeks and birth weight > 1500 g.
8.	Parents and participant understand protocol and are willing to accept randomization to binocular training group or sham-control group.
9.	Patient had not received prior vision training or orthoptics for any reason.
10.	The location of the home address is not very far from the hospital, and if assigned to the training group, training 1–2 times a week at hospital is acceptable.
Exclusion criteria	
1.	Coexisting vertical deviation > 5 PD.
2.	Esodeviation > 5 PD at near or distance
3.	Complaints of diplopia 1 month after surgery in the primary and reading positions.
4.	Limitation of ocular rotations resulting from restrictive or paretic strabismus.
5.	Craniofacial malformations affecting the orbits.
6.	Interocular visual acuity difference more than 0.2 logMAR.
7.	High AC/A ratio (exclude > 6:1 by gradient method).
8.	Prior strabismus surgery or botulinum toxin injection.
9.	Prior intraocular or refractive surgery.
10.	Significant neurological impairment such as cerebral palsy. Participants with mild speech or learning disabilities or both are eligible.

*PACT* simultaneous prism and alternate cover test, *IXT* intermittent exotropia, *D* diopter, *PD* prism diopter, *SER* spherical equivalent refraction, *AC/A* accommodative convergence/accommodation

### Tests performed at baseline and each follow-up visit

After obtaining consent and assent, health-related quality of life (HRQOL) will first be assessed using the Intermittent Exotropia Questionnaire (IXTQ) [25]. It will be completed by each participant and their guardians in separate and quiet rooms. Then, each participant will undergo comprehensive ophthalmic examinations, including best corrected visual acuity (BCVA), subjective refraction, slit-lamp biomicroscopy, and fundus examination. Then, strabismic and binocular visual examinations will be performed, including stereopsis, the amplitude of fusional vergence at far and near, monocular and binocular accommodative amplitude, monocular and binocular accommodative facility, near point of convergence, the prism and alternate cover test (PACT) and simultaneous prism and cover test (SPCT). The examination will first test for binocular and then monocular functions without breaking fusion. If the fusion is broken, for example, the monocular items will be used first for examination. The present study includes a baseline visit and 6-, 12-, 18-, and 24-month follow-up visits. Each examination will be completed on the same day. The 4 follow-up visits will fall into a ± 2-week range. The HRQOL will be completed at the first and last examinations. To ensure the quality and repeatability of data acquisition, we will ask two experienced clinicians to perform all testing procedures; they will be blind to the patient’s randomization status. The standard process regarding the important test items is as follows:

#### Health-related quality of life questionnaire

We will use the Intermittent Exotropia Questionnaire (IXTQ) [25] to assess the participants’ and their guardians’ health-related quality of life (HRQOL). The IXTQ consists of three components:

1. Child questionnaire: It will be used to assess the feelings of the child about his or her eye condition. Since the children will be over 7 years old, they will already have a certain level of reading comprehension. It consists of 12 question items and a five-level response scale (never, almost never, sometimes, often, and almost always). It will be self-administered. However, if some children have hard time understanding the question, we will assign staffs for guidance.
2. Parent proxy questionnaire: This will be used to assess how parents think their child’s eye condition affects their child. It has 12 items and a 5-level response scale.
3. Parental questionnaire: This will be used to assess the parents’ feelings about their child’s eye condition. It has 17 items and a 5-level response scale.

All questionnaires about the parents are completed by the parents themselves. Parents and children will be separated into two different rooms during the completion of the questionnaire to minimize their interaction.

#### Exodeviation control at distance and near

We will assess the exodeviation control at each follow-up period using the Office Control Score [26]. This assessment will be conducted before all other examinations. The investigator will look at the participants’ eyes when they focus at an accommodative target at distance (6 m) or near (40 cm). Their score will be determined as shown below:

- 5 is constant exotropia
- 4 is exotropia > 50% of the 30-s period before dissociation
- 3 is exotropia < 50% of the 30-s period before dissociation
- 2 is no exotropia unless dissociated; recovers in > 5 s
- 1 is no exotropia unless dissociated; recovers in 1–5 s
- 0 is no exotropia unless dissociated; recovers in < 1 s (phoria).

#### Stereoacuity testing

Near and distance stereoacuity will be assessed using the Titmus circles test and TNO at 40 cm and the Distance Randot Stereotest at 3 m, respectively. Stereoacuity will be assessed with the participant’s refractive correction. The Titmus circles will measure from 40 to 800 s of arc (arcsec). The TNO test (Laméris Ootech B.V., Nieuwegein, the Netherlands) will range from 15 to 480 arcsec. The Distance Randot Stereotest (DRS; American Stereo Optical Company U.S.A.) ranges from 63 to 400 arcsec. Testing will start with the largest disparity, and the inability to correctly identify the target with the largest disparity will be recorded as nil stereo. Nil stereoacuity will be assigned the next highest log level (near 1600 s of arc for Titmus; 960 s of arc for TNO; distance 800 s of arc for DRS) for our data analysis.

#### Sensory fusion

The Worth 4 Dot test will be conducted at a 40-cm and 5-m fixation for the assessment of near and far sensory fusion, respectively. The participants will wear red-and-green spectacles and view the Worth 4 Dot flashlight at near and at distance. If participants report 2 red or 3 green lights, it will be taken to represent “suppression” of the left eye or right eye. If they report 4 lights at distance or at near, it means that they have a “normal” peripheral or central fusion. If 5 lights are reported, it will represent “diplopia”.

#### Fusional vergence

The amplitude of fusional vergence will be assessed by a 1 to 40 PD prism bar for both near and far fixation. Each participant will fixate on a single letter (20/40 Snellen level) at 33 cm or 5 m and will be asked to report when the fixated letter appears to double (this will be recorded as the break point) as the prism strength gradually increases. Then, the prism power will be gradually decreased, and the point at which the patient regains single vision will be recorded as the recovery point. Participants will be reminded to keep the target single as long as possible. Both the divergence (BI) and convergence (BO) of the break point and recovery point will be recorded at near and at distance. If the subject reports suppression, which will be assessed by the Worth 4 Dot test, then this part will be omitted and recorded as 0/0. For subjects who still retain fusion at 40 PD, the break point will be recorded as 40 PD, and the recovery point will be recorded as “-” and excluded from the recovery analysis. The fusional vergence at distance will be first and then the vengeance near (divergence and then convergence). The PD values of break points will be defined as the amplitude of the vergence, which will be defined as convergence reserve in our study.

#### Near point of convergence

For this test, participants will fixate at a single Snellen 6/12 letter as an accommodation target at 40 cm in front of their eyes. The investigator will move the target closer to the subject and observe the subject’s eyes until he or she reports diplopia or one eye drifts outward. This point will be recorded, and the distance from the point to the canthus or the plane of the subject’s glasses will be measured.

#### Accommodative function

The accommodative function that we will measure here will include accommodative amplitude and facility. We will separately measure binocular and monocular function in both examinations. Generally, we will measure accommodative facility first and then amplitude, and we will measure binocular function first then monocular function. When measuring binocular function, we will observe the subjects’ eye position and remind them to maintain binocular single vision. The test will be aborted if the patient has diplopia or an outward deviation.

We will use standard test methods (± 2.00 D flipper lenses) to test this facility. The subjects will be asked to read one line above the best visual acuity at 40 cm with their corrected lenses. Then, the + 2.00 D lenses will be placed in front of the subjects’ eyes, and when the letters are reported to be clear, the flipper (− 2.00 D lens) will be quickly flipped in front of the eyes; 1 cycle is achieved when clear is reported again. This will be continued while the flipper lenses alternate for 1 min, and the cycles achieved will be recorded.

We will use “minus lens” to test the amplitude. A near target will be set at 40 cm, and minus lenses will be gradually added in front of the subjects’ eyes until they report sustained blur. The summation of the number of minus lenses and 2.50 D for working distance will provide the total accommodative amplitude.

#### Ocular alignment testing

Ocular alignment will be assessed by SPCT and PACT both at near (33 cm) and at distance (5 m). The deviation will be recorded as constant or intermittent if a manifest exodeviation is present in at least 3 cover and uncover tests. The amount of manifest exodeviations will be recorded by the values of SPCT, and the total amount of exodeviations will be recorded by the measurement of PACT. If no deviation is present at any time, it will be recorded as “no deviation”.

### Assignment of interventions: allocation

#### Sequence generation

A biostatistician will generate a simple randomized number list at a ratio of 1:1 using SPSS software version 20.0 (IBM Corp., Armonk, NY, USA). Patients will be randomly assigned through a computer-generated randomization program. Each participant will be provided a unique ID. Once they meet the inclusion criteria, they will be assigned to one of the two groups by a research assistant. If the participant is assigned to the blank control group, he or she will be informed to follow the routine clinical follow-up time, that is, 6, 12, 18, and 24 months; if the participant is assigned to the orthoptic therapy group, he or she will be guided to the orthoptic therapist and be given the detailed therapeutic schedule. If the patient is not satisfied with the group assignment after randomization and does not want to participate in the follow-up study, that patient will be dropped out from the study. All procedures will be performed by two investigators, and they will be blinded to the interventions.

#### Concealment mechanism

The current study design is single-blind, which means that only the investigators will be blinded to the groups. The patients and their parents (or guardians) will sign the informed consent form and then will be randomly assigned to one of the two groups to avoid bias.

#### Implementation

A statistician from our clinical research department, who is not directly involved in the study, will provide a random sequence before the project starts. In addition, neither investigators nor patients would have access to the random sequences.

### Intervention

After a subject completes all of the examinations, the checklist will be handed to our research assistant. Subjects will be randomly divided into Group A (orthoptic therapy group) and Group B (control group) based on the order provided in the random table.

#### Intervention for the orthoptic therapy group

All participants in the orthoptic therapy group will receive at least 8 weeks of hospital-based orthoptic therapy combined with home reinforcement administered by an orthoptic therapist based on their baseline binocular visual status. The therapy protocol in the present study is based on participants’ binocular vision status and modified on the basis of the Convergence Insufficiency Treatment Trial randomized clinical trial [27, 28]. Hospital-based training will mainly include methods that are not easy to master, the operation is more complex, and the training equipment is not suitable for home use. Furthermore, some participants with poor visual function will need to complete this training under the guidance of a therapist. To enhance the treatment confidence of participants and parents, the therapeutic procedures will be arranged from easy to difficult levels. At the same time, therapists will often verbally motivate the participants to perform the more difficult tasks.

The participants will receive hospital-based therapy 1–2 times per week, 60 min each time, combined with practice at home for 15–30 min, 5 days per week. Therapists will also instruct parents to complete the training log and maintain communication through WeChat in an attempt to enhance compliance and improve the therapeutic effect. The overall treatment program will consist of three phases. In each phase, there are a number of subcategories. The detailed therapeutic program is summarized in Table 2. The participants in our study will have normal binocular visual acuity and basic binocular visual function, which may be different from other patients with abnormal visual function. Each procedure has a designated endpoint that should be obtained before moving on to the next level or phase.

**Table 2:** Hospital based and home-based orthoptic therapy procedure

Phase	Therapy location	Training technique	Endpoint
Phase 1	Hospital-based therapy	Training with major amblyoscope to build fine retina correspondence	Achieve simultaneous vision and build fusion
		Cheiroscopic drawing with single oblique stereoscope	Achieve simultaneous vision and build fusion
		Vectograms (quoits or clowns) for convergence	33 BO for twice for patients without stereopsis
		Increasing fusional convergence based on stereoscopic stimuli	34 BO for twice for patient with stereopsis
	Home therapy	Red/green bar reader	Maintain single binocular without suppression
		Barrel card	Easily fuse three dots
		Brock string	Successfully converge to 2.5 cm from his or her nose.
		Letter chart monocular accommodative facility (MAF)	Start from 20/50, go to 20/40 or 20/30, when result of MAF is 17.5 cpm
Phase 2	Hospital-based vision therapy	Training with major amblyoscope to increase fusional convergence	Fusional convergence reserve is more than +25° (based on different subjective angle of exodeviation, the amount of fusional convergence demand is different)
		Vectograms (quoits or clowns) for divergence	15 BI with stereopsis
		Aperture rule for convergence and divergence	12 pictures for convergence, 7 pictures for divergence.
		Random vergence facility based on stereoscopic stimuli	More than 650 times in 15 minutes
	Home-based vision therapy	Aperture rule	7 for divergence and 12 for convergence
		Letter chart BAF	More than 17.5 cpm (20/30)
		Computerized binocular vision therapy procedure	Achieve clear single binocular vision with 40 to 50 BO and 10 to 15 BI.
Phase 3	Hospital-based vision therapy	Training with major amblyoscope to increase fusional convergence	Fusional convergence reserve is more than +25° (based on different subjective angle of strabismus, amount of fusional convergence demand is different)
		Aperture rule with lens or prism flipper	Accomplish with ±2.00 lens flipper
		Random vergence facility based on stereoscopic stimuli	More than 650 times in 15 minutes
		Bernell-O-Scope for vergence facility	8 pictures for BI and BO each
		Free space fusion with lifesaver card	Easily accomplish convergence and divergence
		VR training that combines pursuit and saccade	Accuracy ratio more than 90% in increasing difficulty
	Home-based vision therapy	Aperture rule with ±2.00 lens flipper	Accomplish 7 in divergence and 12 in convergence
		Lifesaver card	Easily accomplish divergence and convergence

*BO* base out, *BI* base in, *BAF* binocular accommodative facility, *cpm* cycles per minute, *VR* virtual reality

Before the start of each training, some simple binocular function tests will be performed, and the training program will be adjusted based on those test results. The patients can stop training in the hospital and maintain home training (15 min each day) to maintain efficiency if the test results showed that performance had reached or even exceeded the normal level. However, if a decrease in the test results is observed during the follow-up, the appropriate therapeutic treatment will be reinitiated. In addition, the home training is a computerized procedure for binocular vision therapy. Also, some will be asked for 1–2 times a week hospital-based training. For the results and purpose of our study, parents will be required to record the training in detail, including time, frequency, and simple training experience (e.g., difficult, easy, unwilling to do).

#### Control group therapy

For the participants in the control group, all binocular visual function tests will also be completely performed at each follow-up visit. If refractive status changes, appropriate prescription will be provided and used in a pair of new glasses.

If only binocular visual function deterioration occurs, it is proposed that they continue to be observed; if accompanied by a small angle of exodeviation but not meeting the criteria of suboptimal surgical outcome or if participants ask for visual function therapy, they will have deviated from the study protocol and will be considered to be dropped out of group. If eye position regression is obvious and the definition of recurrence is reached, he or she will be classified as having a suboptimal surgical outcome. They will be offered a reoperation to align the eye position or start orthoptic therapy during the study.

### Criteria for dropout

The criteria of dropout will be (1) patients in orthoptic therapy group who did not accept training with 2 weeks of enrolment, (2) patients in control group who had started any kind of postoperative non-surgical treatment (e.g., patching, orthoptic training), (3) participants who had requested to be withdrawn from the study. All participants will be permitted to withdraw or terminate the study for any reason during the study, and the reasons will be recorded. Discontinuation or irregular orthoptic therapy will not be a reason for withdrawal from the study, and participants could complete follow-up visits provided if they are willing.

### Patient and public involvement

Before the beginning of this study, we made a survey to the domestic ophthalmologists and optometrists [22]. The survey involved the design of some training program (e.g., time for each training, frequency of hospital training), which facilitated the development of this study. By taking into account of the different learning abilities of patients, we set the difficulty of training program based on the opinions of patients and their parents (or guardians).

### Outcome measurements

Before the beginning of the study, we demonstrated the feasibility of the project, the reliability of the research methods, and the evaluation of the research results. Stereopsis will be checked by the assistant medical staff of the department, and the other binocular vision items and the amount of exodeviation will all be checked by the same senior doctor.

#### Primary outcome

The primary outcomes in our study will be the cumulative proportion of participants meeting suboptimal surgical outcomes at 24 months after surgery; these suboptimal outcomes are defined as (1) exodeviation of ≥ 10 PD at distance or at near using SPCT or (2) loss of 2 octaves or more of stereoacuity from baseline.

#### Secondary outcomes

The secondary outcomes in our study include the following items: the exodeviation at near and distance tested by PACT, fusional convergence amplitude, stereoacuity, accommodative amplitude, and facility.

### Sample size

Sample size calculation is based on the expected value of efficacy from previous studies. The proportion of participants meeting suboptimal surgical outcomes is 16.7% [20] in surgery combined with orthoptic therapy group and 36.5% in surgery alone [21]. We hypothesis that there will be a significant difference in the cumulative proportion of suboptimal surgical outcome at 24 months after surgery between our two groups. We set the statistical power was set at 80% with a type 1 error probability (alpha) of 0.05 based on a 2-tailed chi-square test. The theoretical sample size was 61, with a 1:1 sample ratio. After allowing for a maximum dropout rate of 10% during the 2-year follow-up, we found that the estimated the sample size for each group to reach a statistical significance was 68.

### Data management and monitoring

Work training was held before the start of the project to provide detailed explanation of the trial protocol, standard operating procedures (SOPs) for study operations, and the content of case report forms (CRFs). Investigators will record the results faithfully on the CRFs. Data will be entered in the EpiData 3.1. system. Any incomplete data will be classified as missing or not applicable.

The Clinical Trial Centre Office of the Eye Hospital of Wenzhou Medical University will be responsible for data monitoring and overseeing the whole study. The original data, including private information, baseline data, orthoptic therapy outcome data, follow-up data, and statistical analysis result data will be kept in the Data Monitoring Committee, which is an important department in Clinical Trial Centre Office. The original data set will not be granted to the public. Data will be shared as requested after approval by the Clinical Trial Centre Office. Any relevant publications will be shared with clinical researchers who have interest. Due the relative simple design of the study procedure and a moderate funding, we considered that a formal auditing plan was not required.

### Safety monitoring

To protect the rights and interests of patients, all participants and their guardians will be informed of the potential benefits and possible risks of the trial before they enrol. The whole process will be carried out in a separate and quiet room by a trained professional assistant (JL). After confirming that the patient and guardian understand the relevant content, we will ask both the patient and his or her guardian to sign an informed consent form. If any one of these individuals do not sign the informed consent form, they will not be admitted to the study.

Because the orthoptic therapy being used in our study is a form of functional training, which is a non-invasive operation, the risk of harm to patients is very small. However, we will still inform all individuals of the training process in detail and will give them the researcher’s telephone or WeChat to communicate at any time. If any care is needed post-trial, we will provide professional care service.

### Statistical analysis

Statistical analysis will be performed using SPSS software version 20.0 (IBM Corp., Armonk, NY, USA). The parameters to be calculated are the means ± standard deviations for the continuous variables and the rates (proportions) for the categorical variables.

For the primary analysis, the cumulative proportion of participants meeting the criteria for suboptimal surgical outcomes by 2 years will be compared between the 2 groups using the Kaplan-Meier method. An intergroup difference and a corresponding 95% confidence interval (CI) will also be calculated. Each of the 2 individual components of the specified suboptimal surgical outcome criteria will be assessed using the chi-square test.

For all IXTs who completed the 24-month follow-up, changes in exodeviation measured by PACT, stereoacuity, exotropia control score, accommodative amplitude and facility, and fusional convergence amplitude between baseline and the 24 months will be compared between the two groups in linear regression models that will be adjusted for the corresponding baseline values. Additionally, considering that binocular visual function is different in different age, the final data may be divided into two subgroups according to different ages.

### Dissemination policy

Our findings may have important implications for the public health and could potentially enlighten the research community on refining the standard clinical treatment for IXT. All experience summary and results in the research process of this trial will be shared through academic exchange activities, including presentations at relevant scholarly conferences, continued education training course. The final results will be published in peer-reviewed scientific journals. All those who have contributed to the design and conduction will be eligible for authorship of subsequent publications.

## Discussion

The success rate following IXT surgery has been quite variable across studies, which can be attributed to differences in examination methods, criteria for surgical failure, and length of follow-up [8, 21, 29, 30]. It has been confirmed that there is a high rate of exodrift or recurrence after surgery in IXTs. A previous study has indicated that orthoptic therapy is effective in the treatment of convergence insufficiency IXT with a maximum deviation of 25 PD [17, 18]. It has also been applied as a component of a postoperative combination therapy [21] but in a retrospective small sample study. Therefore, there are still many related unanswered questions, such as which patients will respond to this therapy, how long, and at what frequency does orthoptic therapy have to be performed for maximal efficacy, what is the endpoint of treatment and what is the long-term stability of these results. With our study, we hope to answer several of these questions.

One-month post-surgery is an important follow-up time. Discomfort caused by strabismus operations, such as red and swollen eyes, has basically disappeared. At that time, patients can rotate the eyeball freely and complete the relevant visual function examination. Therefore, the time point chosen for this study was 1 month after the operation, and the patients in the training group began training 1 month after the operation. In addition, our inclusion and exclusion criteria are relatively strict and may exclude many patients in clinical practice, such as those with esophoria, combined with diplopia and vertical deviation, amblyopia and anisometropia. In this manner, the confounding factors in enrolled patients will be minimized, and the results will represent a powerful conclusion regarding the internal validity of this study. We have a detailed and clear therapeutic program for IXTs in the training group, including the establishment of therapeutic stages and therapeutic endpoints for each stage. This hospital-based therapy for one patient requires one orthoptic therapist to assist the training. Home-based training requires recording training logs, and computerized training would allow the real-time detection of training times and intensity. It would be helpful for us to fully capture all aspects of the therapeutic situation of each patient and analyze correlations between therapy cooperation and the actual effects of orthoptic therapy.

There are several limitations in our present study. First, the inclusion and exclusion criteria for this study are relatively strict and may not fully reflect the effect of orthoptic therapy on the exodrift of all postoperative IXTs. Second, this study uses only simple randomization, not a dynamic randomization technique based on the patient’s surgical methods, age, diopter, and other data, considering the weekly operability and our large sample. These effects will be taken into account during our data analysis.

In summary, our study will be the first large-sample, prospective randomized controlled study to confirm the effect of surgery combined with orthoptic therapy in IXTs.

## Trial status

The trial protocol presented here has obtained approval from the Affiliated Eye Hospital of Wenzhou Medical University Review Board (2019-108-K-101) and is aligned with protocol version 2, approved on 10 August 2019. The recruitment started on 20 August 2019 and completed on 17 August 2021. Considering the importance of standardized visual function examination and orthoptic training in our study and hoping that our research can be extended and traced, we wrote this study protocol, even though recruitment has already been completed.

## Data Availability

The ethical approval does not permit to sharing the entire data that we have acquired, but the datasets used and analyzed in our study are available from the corresponding author.
